# Interventions of Brazil's more doctors program through continuing education for Primary Health Care

**DOI:** 10.3389/fpubh.2023.1289280

**Published:** 2024-01-24

**Authors:** Laianny Krizia Maia Pereira, José Adailton da Silva, Ricardo A. de M. Valentim, Thaísa G. F. M. S. Lima, Cristine M. G. Gusmão, Marcela A. da Rocha, Marquiony M. dos Santos, Alexandre R. Caitano, Rosires M. B. de Barros, Tatyana Souza Rosendo

**Affiliations:** ^1^Postgraduate in Family Health, Federal University of Rio Grande do Norte, Natal, Brazil; ^2^Laboratory of Technological Innovation in Health (LAIS), Federal University of Rio Grande do Norte (UFRN), Natal, Brazil; ^3^Department of Biomedical Engineering, Federal University of Rio Grande do Norte (UFRN), Natal, Brazil; ^4^Brazilian Ministry of Health, Brasília, Brazil; ^5^Department of Biomedical Engineering, Federal University of Pernambuco (UFPE), Recife, Brazil

**Keywords:** continuing education, health policies, public health, Primary Health Care, distance education

## Abstract

**Introduction:**

Brazil's More Doctors Program, in its training axis, aims to improve medical training for Primary Health Care through interventions related to the reality of the territory. The research presented here analyzed the interventions implemented by Brazil's More Doctors Program physicians, members of the Family Health Continuing Education Program, and the relationship with Primary Health Care programmatic actions.

**Methodology:**

The research conducted made use of Text and Data Mining and content analysis. In total, 2,159 reports of interventions from 942 final papers were analyzed. The analysis process was composed of the formation of the corpus; exploration of the materials through text mining; and analysis of the results by inference and interpretation.

**Results:**

It was observed that 57% of the physicians worked in the Northeast Region, which was also the region with the most interventions (66.8%). From the analysis of the bigrams, trigrams, and quadrigrams, four constructs were formed: “women's health,” “child health,” “chronic non-communicable diseases,” and “mental health.” Terms related to improving access, quality of care, teamwork, and reception were also present among the N-grams.

**Discussion:**

The interventions carried out are under the programmatic actions recommended by the Brazilian Ministry of Health for Primary Health Care, also addressing cross-cutting aspects such as Reception, Teamwork, Access Improvement, and Quality of Care, which suggests that the training experience in the Family Health Continuing Education Program reflects on the way these professionals act.

## 1 Introduction

The shortage and poor geographic distribution of general practitioners is a global health problem that also affects both developing and developed countries, such as Canada and the United States. Meanwhile, some places have high concentrations of general practitioners only in large urban centers, leading to important inequities in remote areas, such as lack of access, combined with the difficulty of securing a workforce (1–3). In Brazil, the Brazilian National Health System (SUS) has historically faced difficulties in providing and retaining these professionals in Primary Health Care (PHC), especially in the North and Northeast regions, with an insufficient ratio of physicians per inhabitant (4, 5).

Faced with this historical challenge, high demand, and pressure from managers and society, the Brazilian Ministry of Health (MoH) established Brazil's More Doctors Program (PMM) in 2013, based on Law 12,871/2013. The operationalization of the PMM follows three axes: (a) immediately meet the need for doctors in PHC—emergency provision, (b) improve the infrastructure of Health Units and, (c) improve medical training (6, 7).

Throughout its implementation, the PMM has undergone several evaluations, bringing analysis on the significant expansion of medical coverage in municipalities of regions with greater social and economic issues, such as the North and Northeast, reduction of inequality in the distribution of doctors in Brazil or analysis of undergraduate training that detected an expansion and internalization of medical course places in the country, but with characteristics of privatization and concentration in the richest regions (8, 9). The provision of doctors by the PMM has further reduced inequities and improved the work process by performing a greater number of activities related to PHC (10).

There are few analytical approaches to the formative axis of the PMM in the literature. The training axis of this Program and its initiatives related to continuing education are relevant strategies to make PHC more resolutive and inserted in the SUS network, and studies are needed to deepen the analysis of this object within the scope of the PMM (11).

It is important to clarify that in Brazil, continuing education in the context of health refers to a policy that aims to guide the training and qualification of professionals, considering as its object the problems and needs arising from the work process, with a focus on improving the quality of health care and management (12, 13).

The training axis of the PMM was designed to improve medical training for PHC, with the following objectives: (a) to provide greater experience of medical practice during training; (b) to expand the insertion of doctors in training in SUS services; and (c) to strengthen the continuing education policy integrating teaching-service (6, 14).

Thus, the physicians selected to work in this Program should obligatorily participate in improvement activities, always integrating teaching-service activities (15). The continuing education activities of the PMM are structured in two training cycles, the first cycle being characterized by the fulfillment of the Post-graduation in Family Health (Lato Sensu) and the university pedagogical supervision program. The second cycle is characterized by the continuity of learning based on the unique pedagogical needs of each professional, according to the needs presented by their daily work in the health unit, requiring completion of at least 30 (thirty) hours per month, taking into account the sum of all activities of the educational modules taken (16).

To this end, the MoH integrated Brazilian Public Universities, which were responsible for offering postgraduate courses (Lato Sensu), stimulating continuing education guided by the problems of the territory, and aiming to meet local needs and implement enhancements in health services (16).

In 2018, the Family Health Continuing Education Program (PEPSUS) was initiated, offered in the Virtual Learning Environment of the Brazilian Health System (AVASUS) of the MoH, which is an initiative to induce the training and qualification of Primary Health Care Teams on a large scale, through Distance Education. It covers a training itinerary that includes postgraduate studies (Lato Sensu), extension, and improvement, based on the learning process guided by the reality of the territories, inducing the interventions that provide improvement in work processes and in the access and quality of health care (17). Since 2018, PEPSUS has been training the doctors inserted in the PMM through the Postgraduate Program in Family Health, that is, the first training cycle.

In this context, this research aims to answer the following questions: (a) what interventions were performed by physicians in the first training cycle of Brazil's More Doctors Program in the Family Health Continuing Education Program? (b) Which themes were most worked on? (c) What is the relationship between the interventions and the programmatic actions recommended by the Brazilian National Health System? Therefore, the objective was to analyze the interventions implemented by PPM physicians in the first training cycle offered by PEPSUS and the relationship of these interventions with the programmatic areas recommended by Primary Health Care.

## 2 Methodology

This study is linked to the larger project entitled “In-service training and the qualification of the Brazilian National Health System, from the distance education of the Continuing Education Program in Family Health,” which seeks to analyze in-service training and the qualification of SUS, from the distance education of PEPSUS.

This is an exploratory study with a qualitative approach, which used the Text and Data Mining Technique (TDM) (18) and Bardin's content analysis (19).

The primary data source used was the Course Conclusion Papers (TCC) of Brazil's More Doctors Program (PMM), who were students of the Postgraduate Program in Family Health (PEPSUS), from the classes offered at AVASUS between 2018 and 2021.

The doctors' Course Conclusion Papers of PEPSUS contain reports of experiences, elaborated from interventions carried out by doctors and their team. The intervention proposal comes from the recognition of a problem identified as relevant in the Primary Health Unit's local reality (17).

From the 942 course completion papers, 2,159 intervention reports were extracted. Data were also collected from the doctors' places of work (capital and Federation Unit) and the number of interventions per class. To explore the materials, we used text mining (TDM), a technique automated by algorithms using N-grams, with great potential for enabling consistency in the interpretation of text monitoring strategies (18). We chose to use TDM in one of the stages of content analysis, as the integration of these methods in studies with large volumes of data is effective in supporting evaluative research (20). In the case of this research, the large volume of documents to be analyzed stands out, so using techniques based on computer intelligence, such as text mining, was an important tool in the methodological process.

The following steps were carried out for text mining in this research: Database Preparation; Pre-processing; and N-gram Extraction, described below.

### 2.1 Database preparation

Intervention reports were extracted from AVASUS and the databases were organized by one of the study researchers. Initially, to build the databases, the reports went through the anonymization process and were transcribed into Excel spreadsheets. Subsequently, the following information was added: identification code, Course Conclusion Paper title, year, and state where the interventions were implemented. After this step, they were stored digitally, similar to the process carried out by Rocha et al. (18). The texts comprised the set of documents submitted to text mining procedures (pre-processing and N-gram extraction) and then to analytical procedures (19).

### 2.2 Pre-processing

At this stage, the database was standardized, where the texts were saved separately and renamed. Next, the texts were normalized, placing them all in capital letters, removing extra spaces between characters; removing any special characters, symbols, or duplicate words in a row; as well as removing accents and numbers. Then, a dictionary of irrelevant words was used to remove non-significant aspects of the language components.

### 2.3 N-gram extraction

Text mining automatically extracted the word sequences forming N-grams (*N* = 2, 3, 4): Bigrams (two related terms), Trigrams (three related terms), and Quadrigrams (four related terms). The N-grams were formed by applying the word count matrix that statistically measured the importance and frequency of each word in relation to the others contained in the same database. Then, 20 each of the most frequent and important Bigrams, Trigrams, and Quadrigrams were analyzed to identify the constructs. The creation of the database and text mining were carried out between August and November 2022.

In the analysis of N-grams obtained by the text mining technique, the categorization criterion adopted was semantic or thematic categories, which consist of discovering the core meanings of the words (14). Therefore, based on the most frequent bigrams, trigrams, and quadrigrams, the themes and areas most addressed in the interventions were identified, and based on the thematic categories, constructs were developed following the Primary Health Care programmatic areas recommended by the MoH, contained in the Primary Care Notebooks (https://aps.saude.gov.br/biblioteca/index/MQ_=_=/Mg_=_=). The interventions were analyzed based on official documents such as the National Primary Care Policy—PNAB 2017, Primary Care Notebooks, and Law n. 12,871/2023, which establishes Brazil's More Doctors Program (PMM), within the scope of the SUS. Furthermore, the identified constructs were validated by a group of experts on the subject.

The project was approved by the Research Ethics Committee of the Onofre Lopes University Hospital (CEP HUOL) under Certificate of Presentation of Ethical Appreciation (CAAE) 29817419.7.0000.5292 and Opinion Number: 4.229.524.

## 3 Results

### 3.1 Characterization of PEPSUS classes

**Figure 1:**
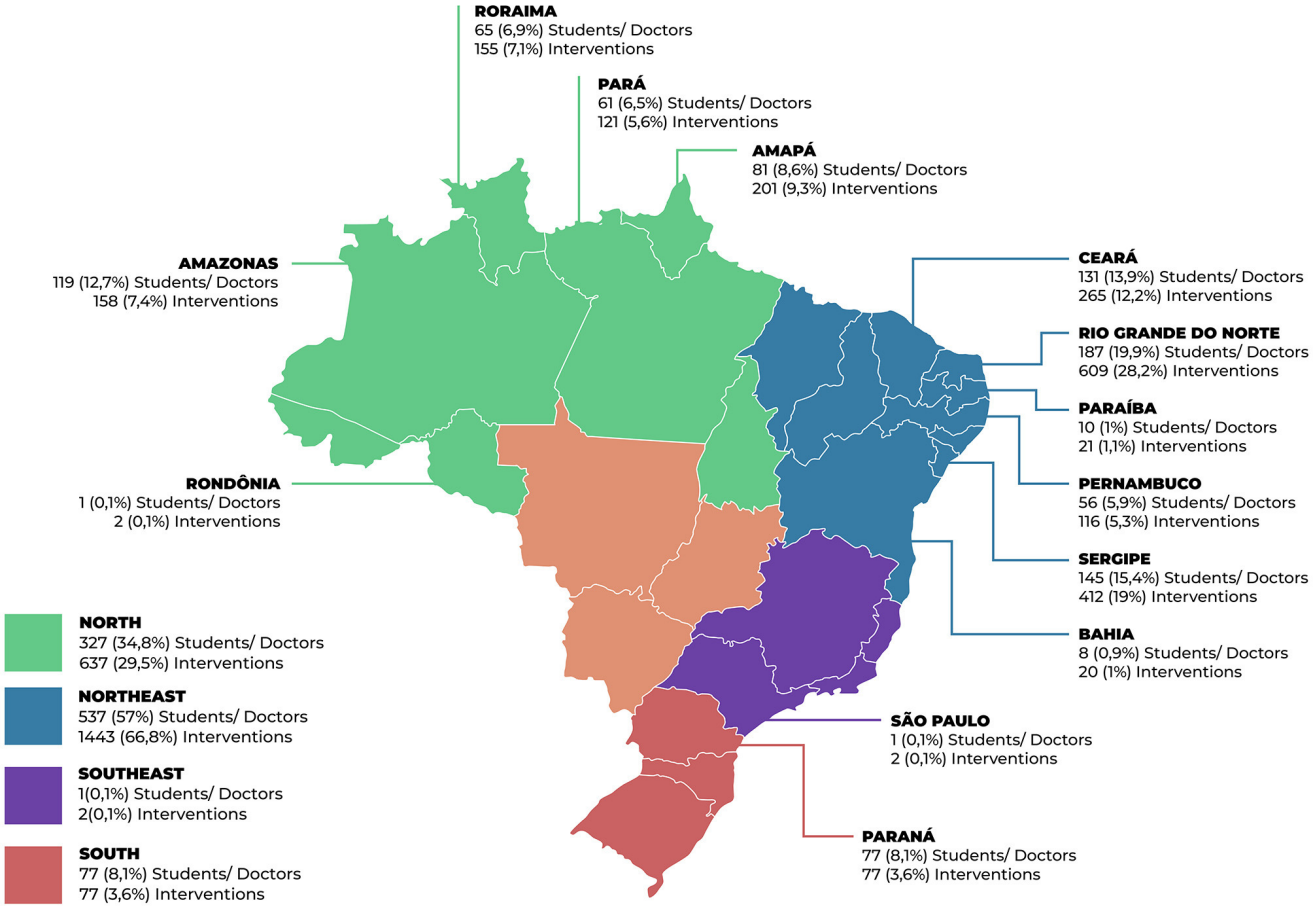
Number of students/doctors and interventions by region and state (6).

Of the total of 942 doctors from Brazil's More Doctors Program (PMM) who completed the Postgraduate Course of the Family Health Continuing Education Program (PEPSUS) between 2018 and 2022, it was observed that more than half worked in the Northeast Region (57%). The states with the highest concentrations of professionals were Rio Grande do Norte (19.9%), Sergipe (15.4%), and Ceará (13.9%) (Figure 1).

A total of 2,159 interventions were carried out, with the highest proportion in the Northeast (66.8%), also highlighting Rio Grande do Norte, Sergipe, and Ceará (Figure 1).

### 3.2 Characterization of interventions carried out by doctors from the more doctors program, according to the approached themes (2018–2021)

**Figure 2:**
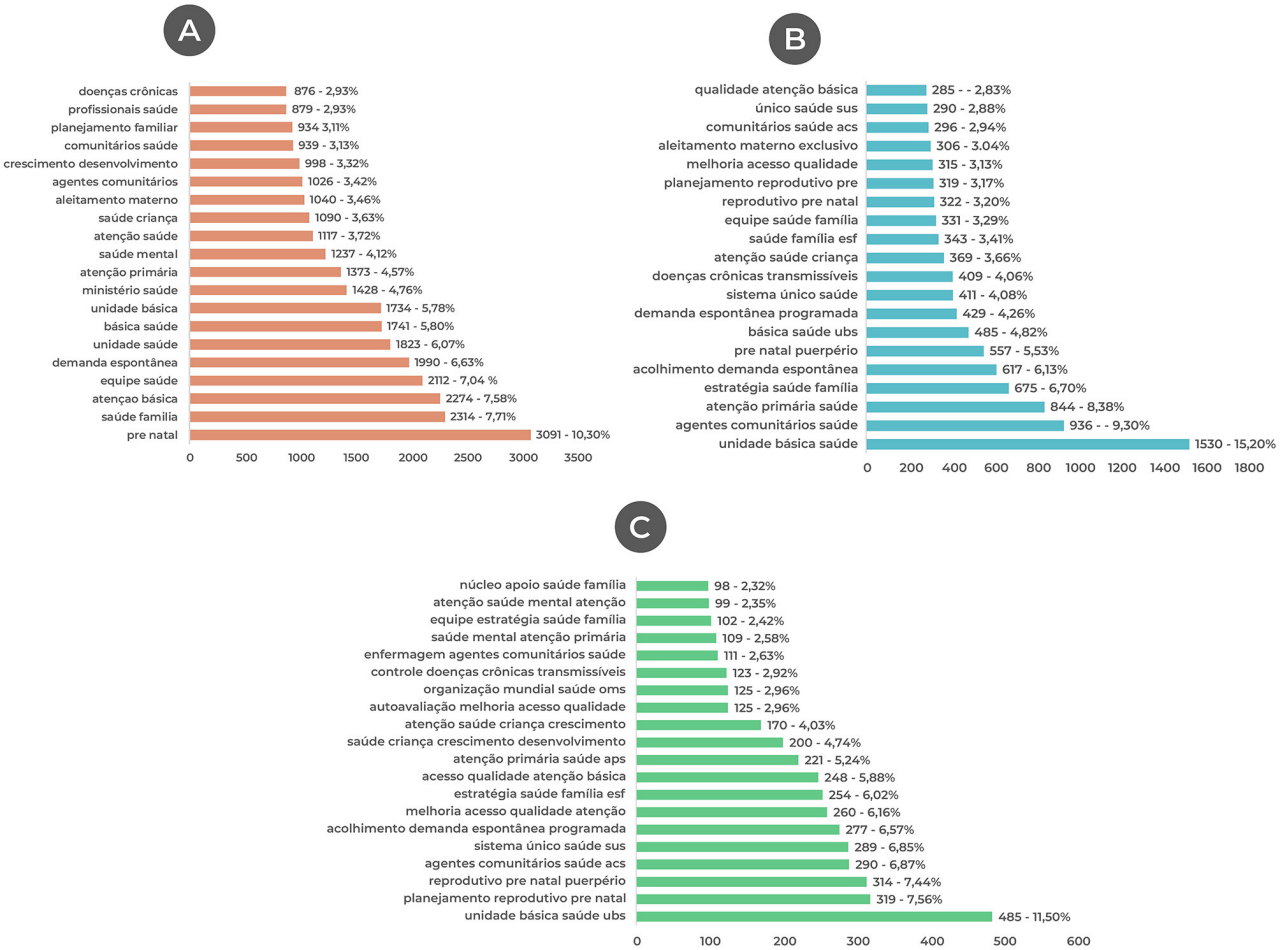
The most frequent terms in the reports of the interventions: **(A)** The 20 most frequent Bigrams in the reports of the interventions; **(B)** The 20 most frequent Trigrams in the reports of the interventions; **(C)** The 20 most frequent Quadrigrams in the reports of the interventions.

The automatic extraction of words, from text mining, resulted in the grouping of the most frequent terms into Bigrams (two related terms), Trigrams (three related terms), and Quadrigrams (four related terms). These groupings, based on the frequency and importance of terms, made it possible to identify the themes most discussed in the interventions. Figure 2 shows the distribution of the 60 most frequent associations of terms in the reports in Bigrams, Trigrams, and Quadrigrams.

The Bigrams showed that the terms “prenatal care,” “family health,” and “basic care” as the three most frequent. The bigrams “health team” and “spontaneous demand” were the fourth and fifth most frequent. Other terms appeared among the 10 bigrams, such as “basic unit,” and “primary care,” which was to be expected given that the interventions had to be developed in the Basic Health Unit and the students' work areas. The bigrams “mental health,” “child health,” and “growth and development” were fewer in number. There were also terms related to teamwork such as: “community health worker,” “health professionals,” and “community workers.” Likewise, it was possible to identify the term “chronic diseases,” which was the least frequent among the 20 bigrams (Figure 2A).

Concerning the trigrams, the most prominent terms were “basic health unit,” “community health workers,” “Primary Health Care,” and “family health strategy,” corresponding to the four most frequent, indicating that the interventions were indeed carried out in primary care, now emphasizing the Community Health Worker. The trigram “welcoming spontaneous demand” appears as the fifth most relevant and “scheduled demand” as the eighth most frequent, indicating interventions aimed at user access. The term “prenatal puerperium” was the sixth most cited trigram. The terms “chronic non-communicable diseases” and “child health care” appeared 10th and 11th, respectively. The trigrams “prenatal reproductive planning” and “prenatal reproductive planning” were also among the top 20 (Figure 2B).

Figure 2C shows the analysis of the quadrigrams, with the term “Primary Care (PC)” having the highest frequency. The second and third most frequent terms are “prenatal reproductive planning” and “puerperal prenatal reproductive planning.” The quadrigram “community health worker (CHW)” was the fourth most common term in the reports. The quadrigrams “welcoming spontaneous demand,” “improving access to quality care,” and “access to quality primary care” appear among the 10 most frequent. The terms “child health growth and development,” “child health care growth and development,” “chronic communicable disease control,” and “mental health primary care” were cited among the 20 quadrigrams presented.

### 3.3 Constructs drawn from the most frequent themes presented in the bigrams, trigrams, and quadrigrams and the analysis of the programmatic areas of Primary Health Care

**Figure 3:**
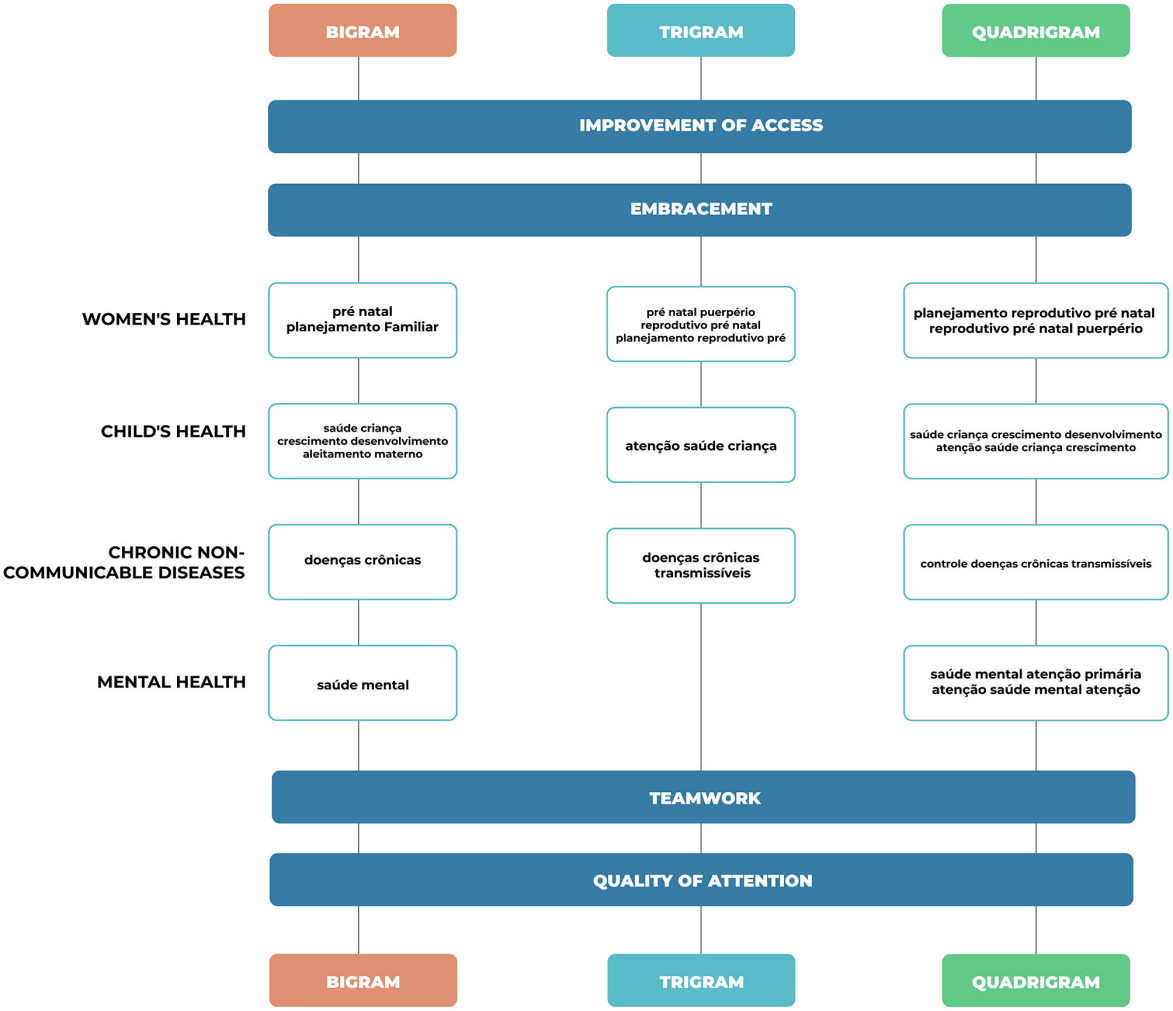
Constructs based on the bigrams, trigrams, and quadrigrams related to the Primary Health Care program areas most present in the interventions.

Figure 3 presents the constructs formed from the analysis of N-grams and the PHC programmatic areas recommended by the Brazilian Ministry of Health. Four constructs relating to four programmatic areas were identified: “Women's Health” (which includes prenatal care, puerperium, and sexual and reproductive health), “Children's Health,” “Chronic Non-Communicable Diseases” (hypertension and diabetes), and “Mental Health.” In addition, there were terms related to cross-cutting themes that cut across all the program areas and were grouped into four other constructs, such as: “Improving Access,” “Quality of Care,” “Teamwork,” and “Welcoming.”

To identify the construct of the area related to Women's Health, the frequency of terms such as: “prenatal care,” “family planning,” “prenatal puerperium,” “prenatal reproductive planning,” and “prenatal puerperium reproductive planning,” pointing out that these activities are part of the Women's Health programmatic area, corresponding to the one most addressed in the interventions. These terms, when added together, correspond to a percentage of 13.41% for bigrams, 14.94% for trigrams, and 15% for quadrigrams.

For Child Health, the following terms were found: “child health,” “growth and development,” “child health care,” “child health growth and development,” and “child health care growth and development,” which correspond to the second most worked on the area in the interventions, with 10.41% for the bigrams, 3.66% for the trigrams and 8.77% for the quadrigrams.

The terms related to the area of Chronic Non-Communicable Diseases, which corresponds to the third most present area in the interventions, are: “chronic diseases,” “chronic non-communicable diseases,” “control of chronic non-communicable diseases,” with the following percentages: 2.92% in bigrams, 4.06% for trigrams and 2.92% in quadrigrams.

Thus, for Mental Health, the terms found were: “mental health,” “mental health primary care,” and “mental health care,” making up 4.12% in the bigrams, and 5.0% for the quadrigrams, with no terms related to this area in the trigrams.

The constructs Improvement of Access and Quality of Care were identified using the terms: “health care;” “improvement of access quality;” “improvement of access to quality of care;” “quality of access to basic care;” “quality of basic care;” “quality of basic care;” “self-assessment improving access quality;” 3.72% for bigrams, 5.93% for trigrams, and 15.0% among quadrigrams.

For Teamwork, the terms found were “health professionals;” “community health;” “community agents;” “health team;” “community health acs;” “family health team;” “community [health] agents;” “family health support center;” “nursing community health agents;” “family health strategy team;” and “community health agents ACS,” corresponding to 16.52% for bigrams, 15.53% for trigrams and, 14.24% among quadrigrams. For the Welcoming construct, the frequency of the terms was observed: “spontaneous demand;” “programmed spontaneous demand;” “spontaneous demand reception;” “programmed spontaneous demand reception,” representing 6.83% among bigrams, 10.39% among trigrams, and 6.57 among quadrigrams.

## 4 Discussion

The interventions implemented by doctors from the More Doctors Program (PMM) of the Permanent Education Program in Family Health (PEPSUS) were related to four programmatic areas of Primary Health Care (PHC) recommended by the Ministry of Health in Brazil: Women's Health, Children's Health, Chronic Non-Communicable Diseases, and Mental Health.

Furthermore, the analysis based on the frequency and relevance of terms using N-grams allowed us to identify actions related to transversal themes, which permeate all programmatic areas, which were grouped into four other constructs: Reception, Teamwork, Access Improvement, and Quality of Care. Primary Health Care (PHC) must ensure citizens have access to health promotion, protection, care, and recovery actions in an integrated manner, in accordance with legal precepts (21). This study identified essential transversal aspects of PHC, such as reception and teamwork, which may have contributed to carrying out interventions, and seeking to improve access and quality of care.

In PHC, welcoming is a powerful device for qualifying the care model, as it enables the health team to rethink its work process and make universal access and comprehensive care a reality (22). It is strategic to have identified this construct in the interventions, since valuing welcoming actions allows for the restructuring and reorganization of health practices, taking into account the real needs of users for qualified and resolutive care (23).

The findings of this study point to teamwork. Considering the integrality of care, teamwork can be one of the main strategies for an effective change in work processes, as opposed to the hegemonic medical model centered on the medical professional (24).

Improving access and the quality of care were also themes mentioned in the reports on the interventions. These aspects were probably mentioned due to the influence of Brazil's National Program for Improving Primary Care Access and Quality (PMAQ), implemented in 2011 by the Brazilian Ministry of Health, aimed to induce the expansion of PHC access and quality. There was, therefore, a context of encouraging quality improvement, in which the teams joined the program voluntarily, and the financial incentive was passed on to the municipalities according to the performance achieved (25, 26).

Regarding the characterization of the classes, it was observed that most of the PMM doctors who completed the postgraduate course worked in the Northeast and North regions of the country, with Rio Grande do Norte being the state with the largest number of participants and consequently interventions. This distribution of doctors was probably influenced by the fact that PEPSUS is offered by the Federal University of Rio Grande do Norte (UFRN). Even though it is not the only postgraduate course (Lato Sensu) offered to PMM doctors in Brazil, its reach to doctors from the most vulnerable regions of the country stands out, assuming a strategic role in health training. Doctor provision programs in other parts of the world direct their efforts to the most distant and vulnerable regions, where in addition to financial incentives and professional support strategies, continuing education strategies serve as an incentive in recruiting and retaining professionals for clinically unfavorable locations (27–29).

In terms of the Primary Health Care program areas covered by the interventions, Women's Health was the area that received the most interventions, present in all the N-gram analyses. As the interventions were defined on the basis of the diagnoses carried out in the territories, it can be seen that actions in prenatal care, puerperium, and sexual and reproductive health presented opportunities for improving care in PHC. Low-risk prenatal care stands out as an important action carried out in the context of PHC, which, when it develops qualified actions, contributes to reducing maternal mortality (30). For the new agenda of targets of the Sustainable Development Goals (SDGs) 2015–2030, Brazil has made a commitment to reduce maternal death to < 30/100,000 Live Births (31).

Still, in the “Women's Health” construct, postpartum care, also present in the interventions, points to the need to expand women's health care in the postpartum period. Studies indicate that assistance actions in the postpartum period are still very focused on the care of newborns, demonstrating the need for qualification of the postpartum period also centered on women (32). Sexual and reproductive, also present in interventions, health care is a priority area of action in PHC, essential for complying with guidelines that ensure sexual and reproductive rights, contributing to improving the quality of life and health of individuals (33).

Child Health, the second most frequent area in interventions, is a priority area for action within the Brazilian National Health System (SUS) since infant mortality is an indicator of the population's health conditions and quality of life (34). For the SDG, Brazil must reduce neonatal mortality to a maximum of five per 1,000 live births and under-five mortality to a maximum of eight per 1,000 live births by 2030 (31).

With the increase in doctors coming from the PMM, there was a success in terms of child health, justified by the increase in consultations for children under 1 year old and the increase in the prevalence of exclusive breastfeeding in the first 6 months of life (35). The child population is a priority group covered by various health policies, given their vulnerability to the effects of poverty and lack of access to basic services, reinforcing the need for more strategies to reduce this inequality (36).

The Chronic Noncommunicable Diseases (CNCD) program area, which in this study includes Diabetes Mellitus (DM) and Systemic Arterial Hypertension (SAH), was also pointed out in the reports of the interventions. As far as PHC is concerned, promoting adherence and lifestyle changes are important factors in controlling and managing SAH and DM. However, implementing strategies that encourage users to take responsibility for their self-care is an ongoing challenge, highlighting the importance of interventions aimed at this programmatic area (37).

Mental Health was the program area least identified in the interventions. However, this finding points to the expansion of activities carried out in PHC, providing a strategic space for mental health actions, with reception as one of the most powerful devices for identifying situations related to users' mental suffering. The inclusion of actions aimed at mental health in PHC points to the expansion of care (38).

It should be noted that the study's limitations are related to secondary data usage, making it difficult to deepen the analysis, such as investigating whether there was continuity in the interventions carried out, as we do not have follow-up data. The choice to use N-grams with the extraction of the 20 most frequent and relevant terms may have limited the range of diversity of interventions carried out. On the other hand, despite its limitations, this study was able to present the PHC program areas that received the most interventions, with significant coverage of the Brazilian territory.

The interventions carried out by doctors from the More Doctors Program are related to the programmatic areas of Women's Health, Children's Health, Chronic Noncommunicable Diseases, and Mental Health, and were all recommended by the Ministry of Health for Primary Health Care (PHC). Furthermore, cross-cutting themes such as Reception, Teamwork, Access Improvement, and Quality of Care were identified, which suggests that the training experience in the Family Health Continuing Education Program (PEPSUS) reflected in the way these professionals work. It is necessary to monitor the effects of interventions on the teams' work processes and the sustainability of actions in future studies.

## Data availability statement

The original contributions presented in the study are included in the article/supplementary material, further inquiries can be directed to the corresponding author.

## Ethics statement

The studies involving humans were approved by the project was approved by the Research Ethics Committee of the Onofre Lopes University Hospital (CEP HUOL) under Certificate of Presentation of Ethical Appreciation (CAAE) 29817419.7.0000.5292 and Opinion Number: 4.229.524. The studies were conducted in accordance with the local legislation and institutional requirements. The participants provided their written informed consent to participate in this study.

## Author contributions

LP: Conceptualization, Formal analysis, Investigation, Methodology, Writing – original draft, Writing – review & editing. JS: Conceptualization, Formal analysis, Methodology, Writing – original draft, Writing – review & editing. RV: Methodology, Writing – review & editing. TL: Conceptualization, Methodology, Writing – review & editing. CG: Conceptualization, Methodology, Writing – review & editing. MR: Data curation, Methodology, Software, Writing – review & editing. MS: Conceptualization, Methodology, Writing – review & editing. AC: Writing – review & editing. RB: Writing – review & editing. TR: Conceptualization, Formal analysis, Methodology, Supervision, Writing – review & editing.
